# ERF3 of *Myricaria laxiflora* contributes to waterlogging tolerance by regulating ethylene biosynthesis through transcriptional regulation of *MylACO1*

**DOI:** 10.3389/fpls.2026.1851263

**Published:** 2026-07-30

**Authors:** Linbao Li, Lin Meng, Yueran Tong, Weibo Xiang, Yang Su, Haibo Zhang, Junchen Wang, Bicheng Dun, Chunlong Li, Guiyun Huang, Di Wu

**Affiliations:** 1 Yangtze River Biodiversity Research Centre, China Three Gorges Corporation, Wuhan, China; 2 Hubei Key Laboratory of Rare Resource Plants in Three Gorges Reservoir Area, Yichang, China; 3 National Engineering Research Center of Eco-Environment Protection for Yangtze River Economic Belt, China Three Gorges Corporation, Wuhan, China; 4 State Key Laboratory of Plant Diversity and Specialty Crops, Wuhan Botanical Garden, Chinese Academy of Sciences, Wuhan, China; 5 School of Life Sciences, Lanzhou University, Lanzhou, China; 6 National Key Laboratory for Germplasm Innovation and Utilization of Horticultural Crops, College of Horticulture and Forestry Sciences, Huazhong Agricultural University, Wuhan, China

**Keywords:** ACO, AP2/ERF, ethylene, *Myricaria laxiflora*, waterlogging tolerance

## Abstract

Ethylene-responsive factors (ERFs) act as pivotal regulators during plant responses to waterlogging, but the specific molecular pathways orchestrating flood adaptation in woody species are still not fully comprehended. Here, we functionally characterized MylERF3, a nucleus-localized AP2/ERF transcription factor from the highly flood-tolerant shrub *Myricaria laxiflora*. Expression profiling revealed that *MylERF3* is rapidly induced by waterlogging stress. Overexpression of *MylERF3* in *Arabidopsis thaliana* markedly enhanced tolerance to waterlogged conditions. Further metabolic and transcriptomic analyses indicated that waterlogging stimulates ethylene emission in *M. laxiflora*, a process fundamentally driven by the induced expression of *MylACO1*, which encodes a crucial ethylene biosynthesis enzyme. MylERF3 directly targets and activates *MylACO1* transcription by binding within its promoter. Consistently, *MylERF3* transgenic lines exhibited elevated levels of ethylene production and ACO enzyme activity, along with up-regulation of ethylene biosynthesis-related genes. Taken together, these results demonstrate that MylERF3 functions positively in waterlogging tolerance by modulation of ethylene biosynthesis via regulating *MylACO1*, thereby offering new insights into the transcriptional regulation of flood adaptation in woody plants.

## 1 Introduction

Frequent waterlogging caused by climate change poses a major threat to global agriculture (Hirabayashi et al., 2013). When soil is flooded, roots lose access to oxygen, which inhibits mitochondrial respiration. As a result, plants rely on anaerobic fermentation to cope with the low-oxygen conditions. This alternative metabolism, however, limits energy supply and causes reactive oxygen species (ROS) accumulation, leading to cellular injury (Xu et al., 2006; Daniel and Hartman, 2024). Unlike naturally adapted wetland species, the majority of agricultural crops lack effective mechanisms to cope with prolonged waterlogging (Sasidharan et al., 2018). Consequently, a comprehensive understanding of the molecular mechanisms underlying flood tolerance serves as a fundamental prerequisite for improving the resilience of susceptible crops.

As a rare riparian shrub in the Yangtze River’s Three Gorges area, *Myricaria laxiflora* can survive up to five months of total underwater waterlogging (Li et al., 2024; Wang et al., 2024), making it an ideal model system for uncovering unique flood-tolerance mechanisms that are absent in most agricultural crops. Unlike herbaceous model plants such as *Arabidopsis* and rice, in which most mechanistic studies of waterlogging tolerance have been conducted, *M. laxiflora* is a perennial woody shrub native to the riparian zone, representing one of the few woody species in which flood adaptation can be investigated at the molecular level. Ecologically, this plant plays a key role in protecting riverbanks and maintaining local biodiversity (Chen et al., 2019). Unfortunately, the construction of the Three Gorges Dam altered the local water environment and destroyed its habitats, driving the species to endangered status (Chen and Xie, 2009; Chen et al., 2019). The altered hydrological regime, characterized by unnatural fluctuations in water levels, has severely disrupted the plant’s reproductive cycle and population stability. Therefore, elucidating the molecular basis of its extraordinary waterlogging tolerance is not only essential for formulating effective conservation strategies for this endangered species, but also provides valuable genetic resources for improving the waterlogging resilience of susceptible crops.

Under hypoxic conditions, ethylene serves as a major hormone that coordinates adaptations, such as aerenchyma formation, root growth, and ROS scavenging (Alonso and Stepanova, 2004; Müller and Munné-Bosch, 2015; Zhao et al., 2021). The biosynthesis of ethylene is governed by ACC synthase (ACS) and ACC oxidase (ACO). Although ACS mediates the rate-limiting step, ACO directly converts ACC to active ethylene, playing a central role in waterlogging-induced ethylene accumulation (Müller and Munné-Bosch, 2015; Sasidharan and Voesenek, 2015). For example, in the flood-tolerant *Rumex palustris*, waterlogging quickly increases *RpACO* transcription and ACO activity, leading to enhanced ethylene production (Vriezen et al., 1999). In *Arabidopsis*, waterlogging strongly activates ACS genes (Lee et al., 2011). Additionally, waterlogged roots synthesize the precursor ACC and transport it through the xylem to the shoots, where it is oxidized into ethylene to regulate stress responses (Sasidharan and Voesenek, 2015). Nevertheless, the upstream regulatory networks responsible for activating these ethylene synthesis genes under waterlogging stress remain poorly understood.

Abiotic stresses continuously threaten plant growth and agricultural productivity. To survive these adverse conditions, plants have evolved complex adaptive strategies, including dynamic changes in gene expression at the molecular level (Zhu, 2016; Zhang et al., 2022a). Transcription factors (TFs) govern these transcriptional changes, serving as the essential link between stress signal perception and downstream gene activation. Among the diverse TF families in plants, the APETALA2/ethylene-responsive factor (AP2/ERF) superfamily is a major group (Nakano et al., 2006; Licausi et al., 2013). Extensive evidence indicates that AP2/ERF proteins are essential for ethylene-mediated signaling pathways, conferring tolerance to environmental stresses, including waterlogging (Licausi et al., 2013; Wei et al., 2019). Ethylene triggers a signaling cascade culminating in the stabilization and activation of Group VII Ethylene Response Factors (ERF-VIIs), transcription factors that orchestrate hypoxia-responsive gene expression (Licausi et al., 2013; Gasch et al., 2016). *Arabidopsis thaliana* contains five well-characterized ERF-VII members, namely HRE1, HRE2, RAP2.12, RAP2.2, and RAP2.3, which contain conserved MCGGAI/L domains and are degraded via the N-end rule pathway under normoxia but stabilized under hypoxia (Xu et al., 2006; Gasch et al., 2016; Lou et al., 2022). For example, overexpression of the ERF transcription factor *RAP2.2* enhances waterlogging stress tolerance in *Arabidopsis* (Hinz et al., 2010). The maize transcription factor *ZmEREB180* confers waterlogging tolerance through enhanced adventitious root development (Yu et al., 2019). However, the functional roles of most ERF family members in waterlogging tolerance remain incompletely characterized. Furthermore, the downstream target genes through which ERFs orchestrate waterlogging adaptation remain largely unidentified, significantly constraining comprehensive molecular understanding of this stress response mechanism.

Previous transcriptomic profiling of *M. laxiflora* under waterlogging treatment identified waterlogging-inducible genes. Among these, several ERF-annotated genes exhibited differential induction, with *Myl01g01174* (designated as *MylERF3* herein) showing pronounced upregulation. Here, we establish that MylERF3 positively regulates waterlogging tolerance in *M. laxiflora*. Furthermore, MylERF3 enhances ethylene biosynthesis through direct transcriptional activation of *MylACO1*. Collectively, our results demonstrate that *MylERF3* plays a pivotal role in enhancing waterlogging tolerance in *M. laxiflora* by transcriptionally activating *MylACO1*, thereby modulating ethylene biosynthesis.

## 2 Materials and methods

### 2.1 Plant materials and stress treatments

The cuttings from *M. laxiflora* plants were collected at Yanzhiba (Yichang, China) to obtain genetically uniform experimental material. All plant materials were subsequently propagated and cultivated under controlled environmental conditions at the Experimental Farm of the Rare Plants Research Institute of the Yangtze River. For waterlogging treatment, one-year-old seedlings of uniform developmental stage were selected and transferred to a controlled experimental pond hydrologically connected to the Yangtze River, thereby mimicking natural waterlogging conditions. Throughout the experimental period, the ambient temperature was maintained within a range of 19-27 °C. The waterlogging regime was initiated at 11:00 AM. The root samples were collected at prescribed time intervals (0, 6, 12, 24 and 36 h) following waterlogging initiation, immediately flash-frozen in liquid nitrogen, and stored at -80 °C until subsequent analyses.

For transgenic *Arabidopsis* lines, three-week-old plants were fully submerged in deionized water under dark conditions. The leaves were maintained 10 cm below the water surface throughout the treatment, which began at the start of a 16-h light period and continued through a subsequent 8-h dark phase. Following 48 h of waterlogging, the plants were drained and transferred back to normal growth conditions, with a 16-h light/8-h dark cycle at 22 °C.

### 2.2 RNA extraction and qPCR analysis

Total RNA was isolated from leaf and root samples of *M. laxiflora* and *Arabidopsis* using an RNA purification kit (TIANGEN, Beijing, China). cDNA was synthesized from the extracted RNA using the First Strand cDNA Synthesis Kit (Transgen, Beijing, China). Quantitative real-time PCR (qPCR) amplification was performed using PerfectStart^®^ Green qPCR SuperMix (Transgen, Beijing, China). Gene expression levels, normalized to the Actin reference gene (Xiang et al., 2025), were calculated relative to controls using the 2^–ΔΔCt^ method (Livak and Schmittgen, 2001). Corresponding primer sequences are listed in **Supplementary Table 1**.

### 2.3 Cloning and sequence analysis of MylERF3

The complete coding sequence (CDS) of MylERF3 was amplified from *M. laxiflora* root cDNA using gene-specific primers (**Supplementary Table 1**). Phylogenetic analysis was conducted with MEGA X software, and multiple sequence alignment was performed using GENEDOC to assess sequence conservation.

### 2.4 Subcellular localization analysis

The CDS of MylERF3 was subcloned into the 101LYFP vector, resulting in a MylERF3-YFP translational fusion. Agrobacterium-mediated transient co-expression of the MylERF3-YFP construct (or the 101LYFP control) with a VirD2NLS-mCherry nuclear reference was performed in *Nicotiana benthamiana* leaves, according to the protocol described previously (Meng et al., 2022). Fluorescence signals from YFP (indicating MylERF3 localization) and RFP (nuclear marker) were captured 48 hours post-infiltration using a confocal laser scanning microscope (Leica TCSSP8, Germany).

### 2.5 Transcriptional activation activity analysis

The CDS of MylERF3 was cloned into the pBD vector to generate an effector, while the pGreenII 0800-LUC vector carrying 5×GAL4 activation domain upstream of the CaMV35S promoter was employed as the reporter. The pBD empty vector was used as a negative control, and a pBD construct encoding the LexA DNA-binding domain fused to the VP16 activation domain (pBD-VP16) served as a positive control. All resultant plasmids were individually introduced into *Agrobacterium tumefaciens* strain GV3101, and the transformed Agrobacterium cultures were infiltrated into *N. benthamiana* leaves. Following incubation in darkness at 25 °C for 3 days, luciferase (LUC) activity was measured using the Dual-Luciferase^®^ Reporter Assay System (Promega, Cat. E1910, USA) according to the manufacturer’s protocol. Spatial luminescence imaging was carried out with a NightSHADE LB 985 imaging system (Berthold Technologies, Germany) as previously described (Ming et al., 2021).

### 2.6 Vector construction and plant transformation

The MylERF3 CDS was inserted into the pSAK277 vector for constitutive overexpression. Following verification, the pSAK277-MylERF3 construct was introduced into *Agrobacterium tumefaciens* GV3101. Stable transgenic *Arabidopsis* lines were subsequently generated via the floral dip method as previously described (Clough and Bent, 1998). Stable homozygous T3 transgenic Arabidopsis lines were selected for subsequent phenotypic and physiological evaluations.

### 2.7 Physiological measurement

Electrolyte leakage (EL) was quantified according to established protocols (Zhang et al., 2022b). Briefly, leaf discs were immersed in deionized water and shaken at 25 °C for 2 h to measure initial conductivity (E1). Samples were then boiled at 100 °C for 20 min to release all electrolytes and cooled to determine total conductivity (E2). EL was calculated using the formula: EL = E1/E2×100%. Concurrently, MDA, H_2_O_2_ and O_2_^•-^ levels were measured using respective commercial assay kits (MDA: A003-1; H_2_O_2_: A064-1; O_2_^•-^: A052-1; Nanjing Jiancheng Bioengineering Institute, China), following the manufacturers’ instructions.

### 2.8 Measurement of ethylene production

Ethylene quantification was performed according to a previously described method with minor modifications (Zhao et al., 2014; Zhang et al., 2022b). Briefly, plant samples were placed in 10 mL gas chromatography vials equipped with moistened filter paper to maintain high humidity. Following a 24-hour incubation period at room temperature, 1 mL of headspace gas was sampled from each vial using a syringe and injected into an Agilent Technologies 7890A GC System (Santa Clara, CA, USA) for ethylene quantification.

### 2.9 Measurement of ACO activity

ACO activity was measured as described previously with minor modifications (Bulens et al., 2011). Plant tissues were homogenized in extraction buffer (100 mM Tris pH 7.2, 30 mM sodium ascorbate, 10% glycerol, 5% PVPP) and centrifuged at 4 °C. Then, 200 μL of supernatant was incubated with 1.7 mL of extraction buffer (without PVPP), 100 μL of 1 mM FeSO_4_, and 100 μL of 20 mM ACC in a sealed vial at 30 °C for 1 h. Ethylene production was quantified by gas chromatography as described previously. The activity of ACO enzymes can be calculated on the basis of the amount of ethylene produced.

### 2.10 Yeast one-hybrid assay

The CDS of MylERF3 was inserted into the pGADT7 vector to generate prey constructs. In parallel, promoter fragments (P1) of *MylACO1* containing the conserved DRE/CRT motif were cloned into the pAbAi vector to create bait constructs. Site-directed mutagenesis was employed to substitute the GTCGG motif with TTTTT within the P1 promoter, generating mP1 mutant bait controls. Yeast *Y1HGold* cells were co-transformed with prey/bait pairs (wild-type or mutant) alongside the positive control (pGADT7-p53 + pAbAi-p53). Transformed yeast cells were plated on synthetic dropout medium lacking leucine and uracil (SD/-Leu/-Ura), supplemented with or without aureobasidin A (AbA).

### 2.11 Electrophoretic mobility shift assay

To generate recombinant His-tagged MylERF3, its CDS was ligated into the pHMGWA expression vector. The resulting His-MylERF3 fusion construct was transformed into *Escherichia coli* Rosetta (DE3) competent cells. Recombinant protein expression was induced with 0.5 mM isopropyl β-D-1-thiogalactopyranoside (IPTG) at 16 °C for 16 hours. Target proteins were purified under native conditions using Ni-NTA affinity chromatography (Qiagen, Germany). For EMSA analysis, 40-bp *MylACO1* promoter fragments containing either the wild-type DRE/CRT motif or mutated variants (TTTTT) were commercially synthesized. FAM fluorophore labeling was performed on probe sequences, while unmodified fragments served as cold competitors. Protein-DNA interactions were analyzed via electrophoretic mobility shift assay using the LightShift Chemiluminescent EMSA Kit (Thermo Scientific, Rockford, IL) according to established methodology (Zhang et al., 2022b).

### 2.12 Dual luciferase assay

To generate effector constructs, the MylERF3 CDS was inserted into the pGREEN II-62-SK vector. Reporter constructs were generated by cloning either the native or mutated *MylACO1* promoter sequences into the pGREEN II-0800-LUC vector. All recombinant plasmids were mobilized into *A. tumefaciens* strain GV3101 harboring the p19 silencing suppressor. *N. benthamiana* leaves were co-infiltrated with Agrobacterium suspensions containing effector and reporter constructs. Following 48 hours of transient expression, luciferase (LUC) activity was quantified using the Dual-Luciferase^®^ Reporter Assay System (Promega, Cat. E1910, USA) per manufacturer protocol. Spatial visualization of luminescence was performed using the NightSHADE LB 985 *in vivo* imaging system (Berthold Technologies, Germany) as referenced (Ming et al., 2021).

### 2.13 Statistical analysis

All experimental treatments were independently replicated a minimum of three times. Three independent biological samples were collected and analyzed for each experiment. Data are expressed as means ± standard error (SE). Asterisks indicate statistically significant differences determined by Student’s *t*-test. Significance thresholds were defined as *P* < 0.05 (*) and *P* < 0.01 (**).

## 3 Results

### 3.1 Isolation and expression analyses of *MylERF3*

**Figure 1:**
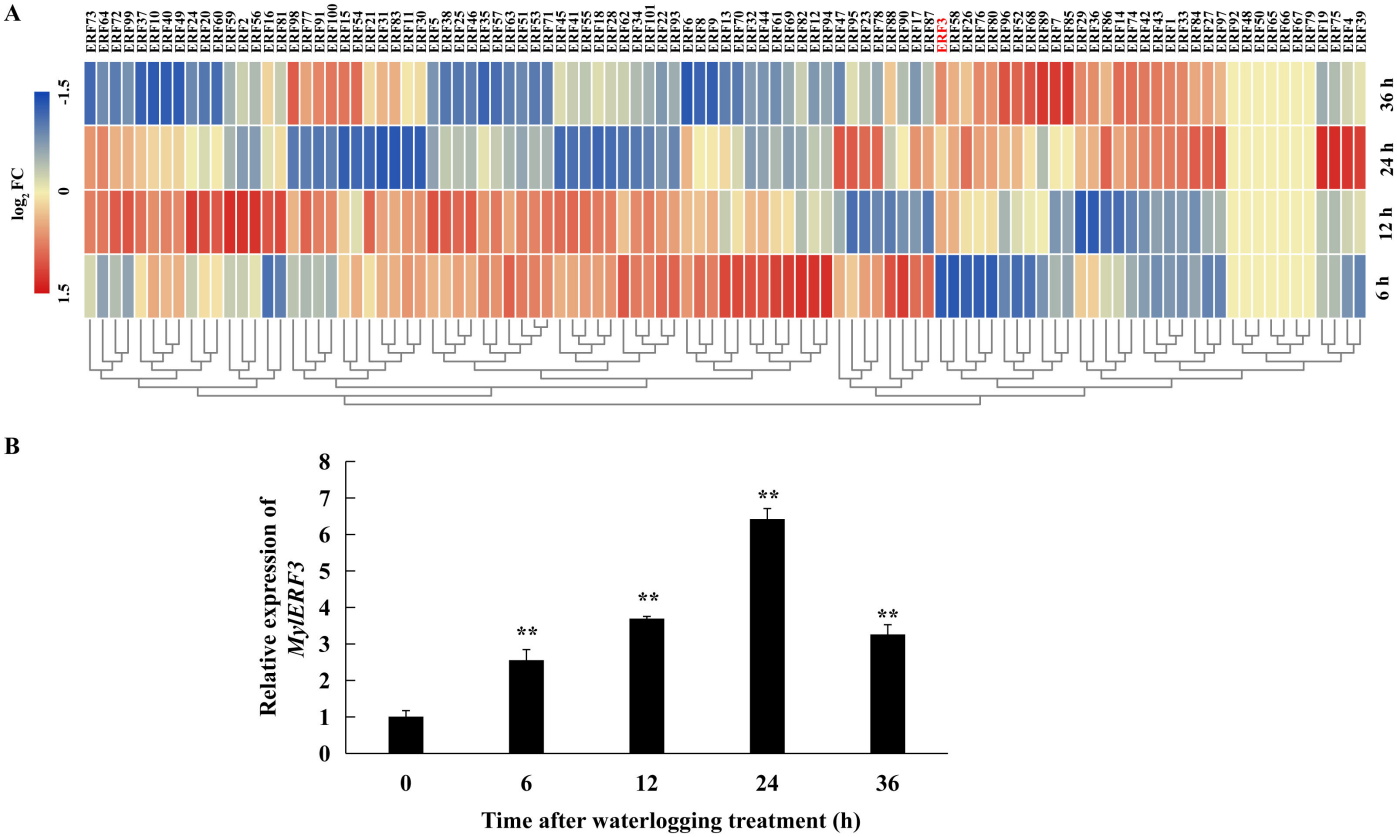
Expression analysis of *ERF* genes under waterlogging treatment in *M. laxiflora*. **(A)** Expression patterns of *ERF* family genes in *M. laxiflora* under waterlogging conditions. **(B)** The relative expression of *MylERF3* in roots under waterlogging treatment. Error bars represent ± standard error (n=3). Asterisks indicate significant differences using Student’s *t*-test (**P < 0.01).

Transcriptional dynamics of ERF family genes in *M. laxiflora* under waterlogging stress were visualized via heatmap clustering, derived from a published RNA-seq dataset (Xiang et al., 2025). Among these, *MylERF3* (*Myl01g01174*) exhibited steady induction under waterlogging stress (**Figure 1A**). The transcriptional dynamics of *MylERF3* under waterlogging stress were analyzed via qPCR. *MylERF3* expression was rapidly induced, exhibiting a 6-fold upregulation at 24 h post-treatment compared to untreated controls (0 h), representing the peak response (**Figure 1B**). The full-length cDNA of MylERF3 was subsequently amplified from waterlogging-treated samples. Sequence data showed that the MylERF3 gene has a 729-bp open reading frame (ORF) that codes for a protein of 242 amino acids. The calculated molecular weight of this protein is 25.7 kDa, with a theoretical pI of 5.44. In the phylogenetic tree, MylERF3 clustered together with the *Arabidopsis* AtERF41 (AT5G11590, **Supplementary Figure 1A**), indicating that MylERF3 belongs to the ERF-III subfamily rather than the well-characterized ERF-VII subfamily typically associated with hypoxia responses. Multiple sequence alignment further revealed that MylERF3 shares a highly conserved AP2/ERF DNA-binding domain with AtERF41, featuring canonical YRG and RAYD elements (**Supplementary Figure 1B**). However, significant sequence divergence was observed in the N-terminal and C-terminal regions. This structural architecture suggests that MylERF3 may possess specialized regulatory functions compared to its Arabidopsis orthologs. This classification suggests a potentially distinct regulatory mechanism underlying waterlogging tolerance.

### 3.2 MylERF3 localizes to the nucleus and has transcriptional activation activity

**Figure 2:**
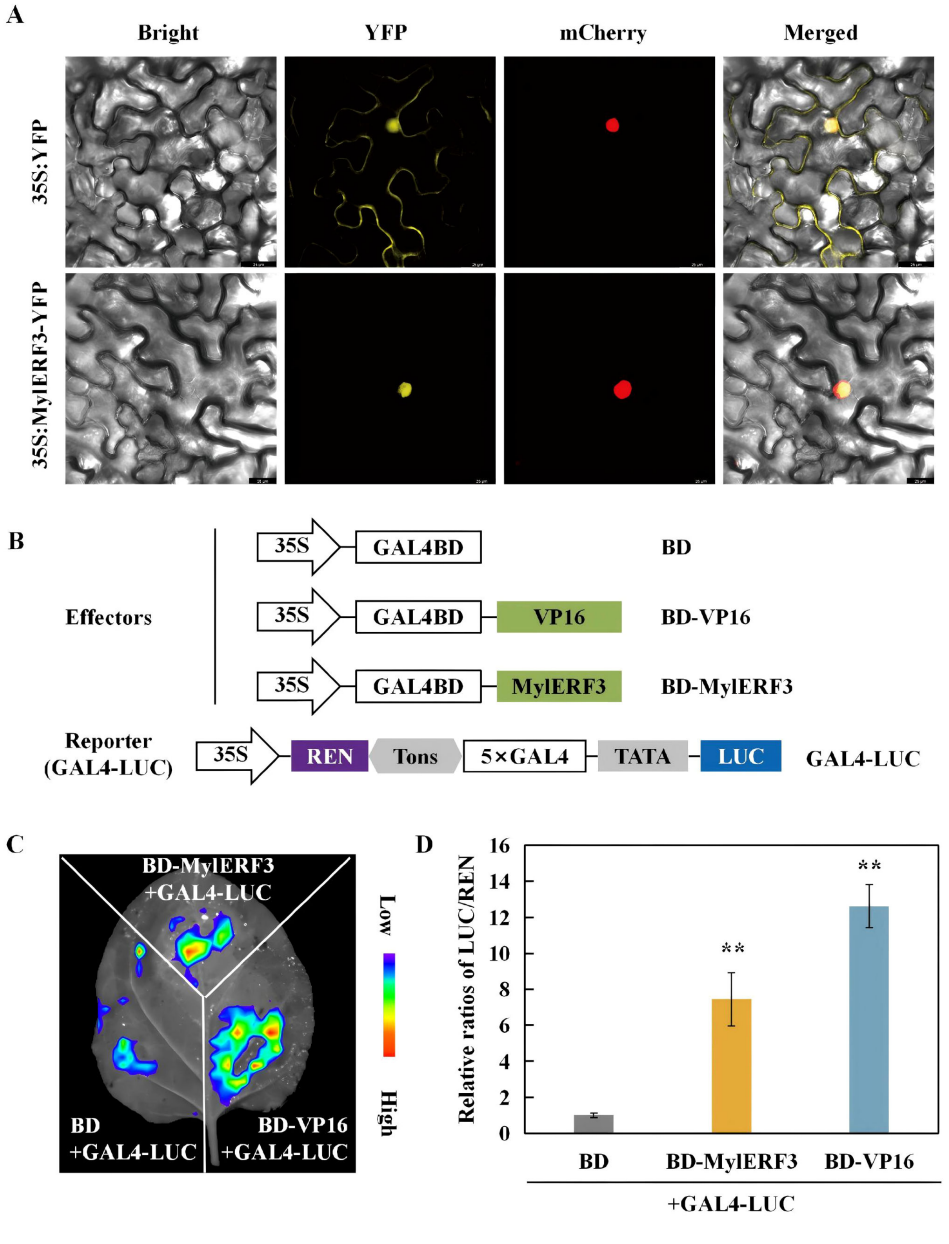
Subcellular localization and transcriptional activation activity of MylERF3. **(A)** Confocal imaging of *N. benthamiana* leaves transiently expressing either the MylERF3-YFP chimera or the unconjugated YFP control. The red fluorescent signal designates the nuclear marks. Scale bars = 10 µm. **(B)** Schematic diagrams of reporter and effector constructs used for transcriptional activation activity analysis of MylERF3 *in vivo*. BD and BD-VP16 were used as negative and positive controls, respectively. **(C, D)** Living image **(C)** and LUC/REN ratio **(D)** in tobacco leaves co-infiltrated with the indicated vectors. Error bars represent ± standard error (n=3). Asterisks indicate significant differences using Student’s *t*-test (**P < 0.01).

To investigate the subcellular targeting of MylERF3, a recombinant construct was generated by fusing the MylERF3 sequence to a YFP reporter gene under the control of the CaMV 35S promoter. This recombinant plasmid, along with a 35S:YFP empty vector control, was transiently expressed in *Nicotiana benthamiana* leaves via Agrobacterium-mediated infiltration. Confocal imaging showed that the fluorescence of the control YFP was present in both the cytoplasm and the nucleus. In contrast, the signal from the MylERF3-YFP fusion protein was restricted to the nucleus, where it completely overlapped with the VirD2NLS-mCherry nuclear marker. These observations directly imply that MylERF3 functions as a nuclear-localized protein (**Figure 2A**).

To determine whether MylERF3 functions as a transcriptional activator, a GAL4-based transactivation assay was performed. MylERF3 was fused to the GAL4 DNA-binding domain and co-expressed with a GAL4-responsive luciferase reporter in tobacco leaves, using VP16 as a positive control and pBD as a negative control (**Figure 2B**). The MylERF3-GAL4 fusion significantly activated reporter gene expression compared with the negative control (**Figures 2C, D**). These results indicate that MylERF3 is a nuclear protein with transcription activation activity.

### 3.3 Overexpression of *MylERF3* improves waterlogging tolerance in *Arabidopsis*

**Figure 3:**
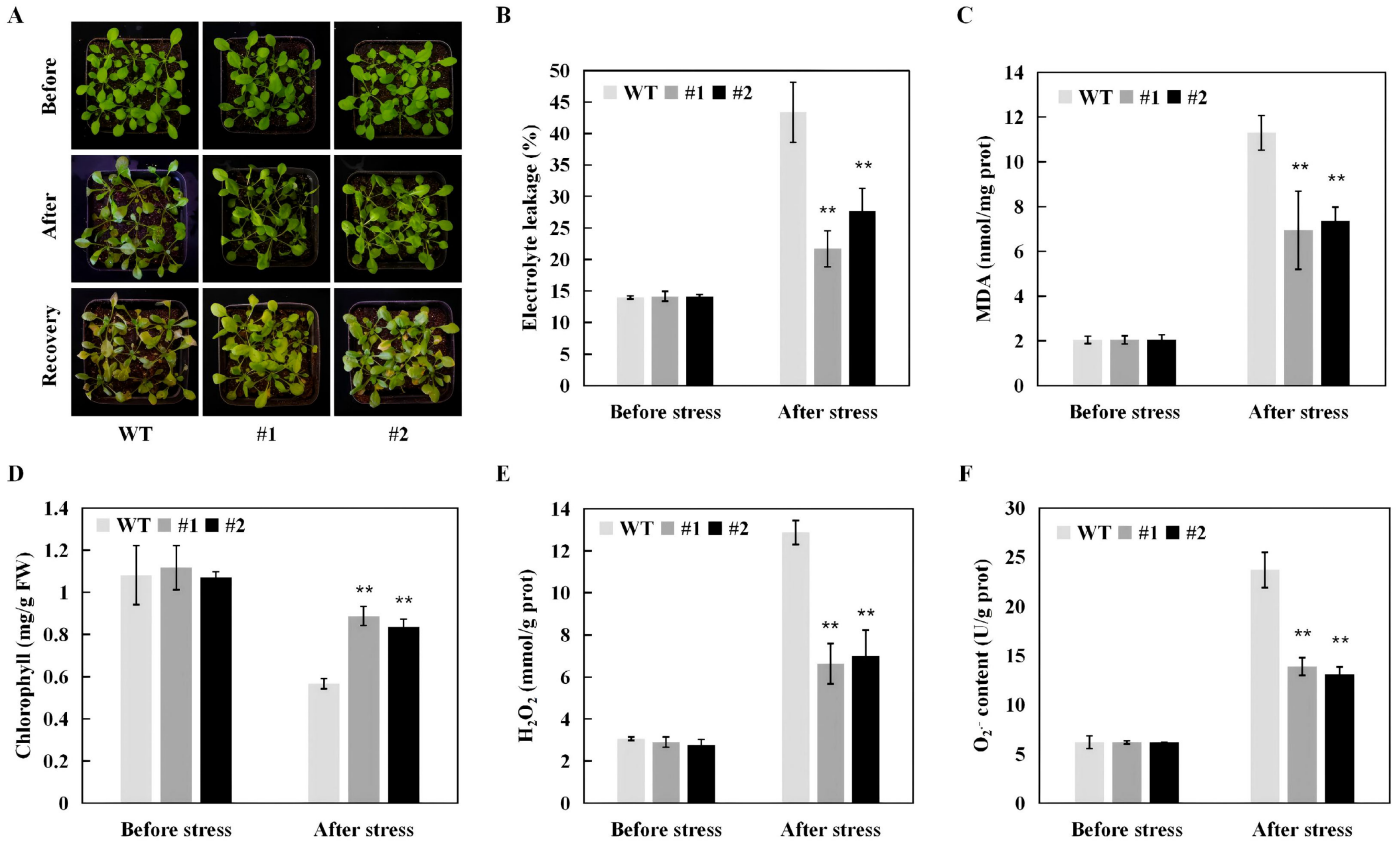
Overexpression of *MylERF3* enhances waterlogging tolerance in *Arabidopsis*. **(A)** Phenotype of wild-type (WT) and two independent *MylERF3*-overexpressing transgenic lines (#1 and #2) after waterlogging treatment. **(B–F)** Electrolyte leakage **(B)**, MDA **(C)**, chlorophyll content **(D)** H_2_O_2_, **(E)** and O_2_^•-^ **(F)** levels in WT and two transgenic lines (#1 and #2). Error bars represent ± SE (n=3). Asterisks indicate significant differences using Student’s *t*-test (**P < 0.01).

Prompted by strong induction of *MylERF3* under flooded conditions, the biological role of *MylERF3* in waterlogging adaptation was investigated. For this purpose, Agrobacterium-mediated transformation was conducted to produce *MylERF3*-overexpressing *Arabidopsis* plants, and two highly expressing lines were chosen for further analysis (**Supplementary Figure 2**). Following extended waterlogging and a recovery period, severe structural impairment occurred in the WT, while the transgenic plants showed much milder symptoms (**Figure 3A**). The significantly lower levels of electrolyte leakage (EL), malondialdehyde (MDA) and ROS (H_2_O_2_ and O_2_^•-^), together with higher chlorophyll content were measured in the transgenic lines compared to the WT (**Figures 3B–F**). Moreover, we analyzed the expression levels of several well-characterized core antioxidant genes, including *AtSOD1* (Kliebenstein et al., 1998), *AtCAT1* (Mhamdi et al., 2010) and *AtAPX1* (Davletova et al., 2005). The RT-qPCR analysis revealed that the transcript levels of *AtSOD1*, *AtCAT1* and *AtAPX1* were significantly up-regulated in the MylERF3-overexpressing lines compared to the WT (**Supplementary Figure 3**). Collectively, these results demonstrate that overexpression of *MylERF3* confers enhanced waterlogging tolerance in transgenic plants.

### 3.4 Waterlogging induces ethylene production in *M. laxiflora*

**Figure 4:**
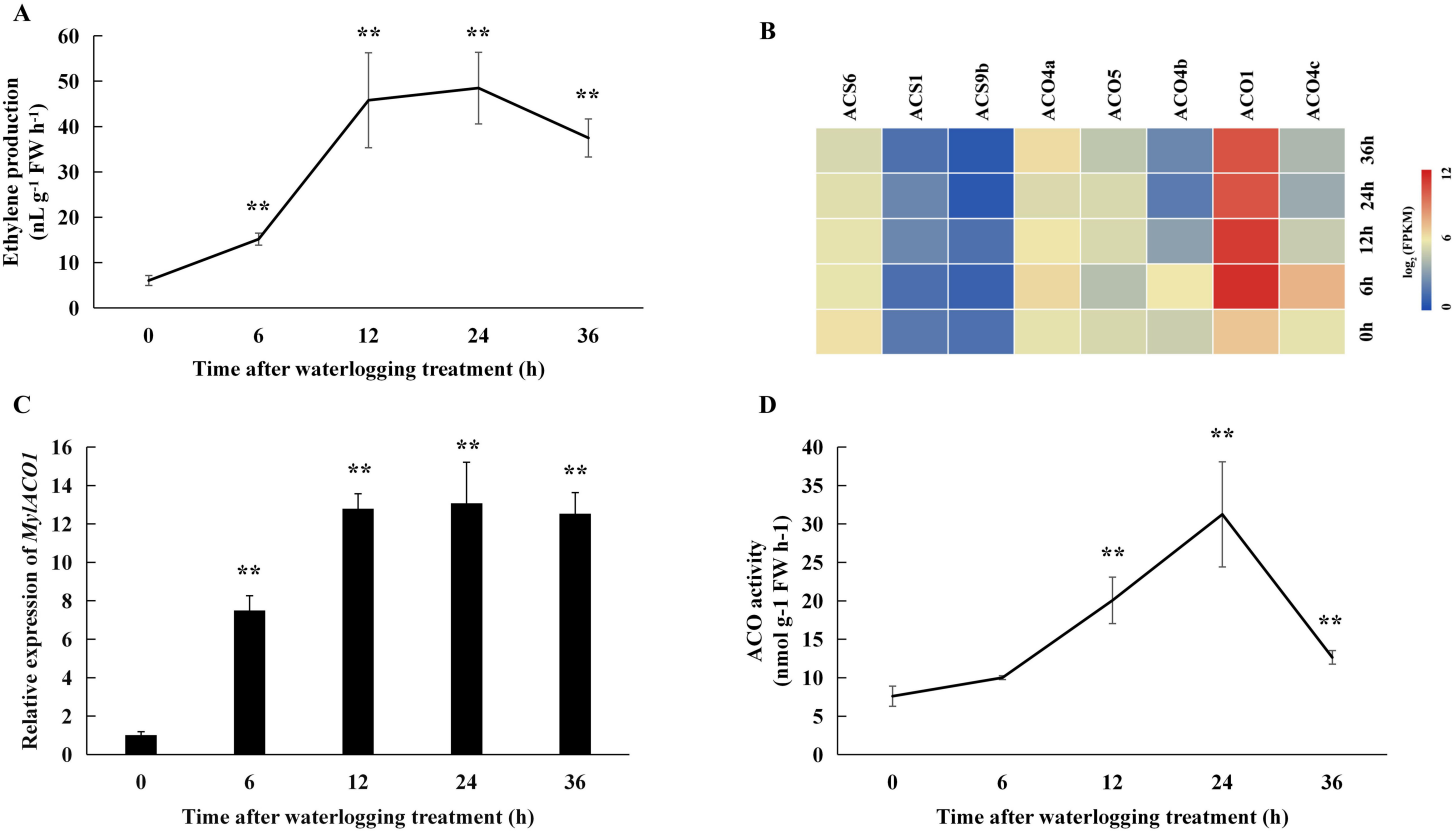
Waterlogging stress induces ethylene production in *M. laxiflora*. **(A)** Ethylene production in roots of *M. laxiflora* under waterlogging stress. **(B)** Expression of ethylene biosynthesis-related genes in waterlogging-treated *M. laxiflora* based on RNA-seq data. **(C)** Relative expression of *MylACO1* in roots quantified by qPCR under waterlogging stress. **(D)** ACO activity in roots of *M. laxiflora* under waterlogging stress. Error bars represent ± standard error (n=3). Asterisks indicate significant differences using Student’s *t*-test (**P < 0.01).

Ethylene serves as a pivotal regulator of plant responses to abiotic stresses, including waterlogging (Alonso and Stepanova, 2004; Momonoi et al., 2007; Sasidharan and Voesenek, 2015). We quantified ethylene dynamics in *M. laxiflora* during waterlogging condition, observing a gradual increase at 6 h followed by a sharp rise to peak accumulation at 24 h (**Figure 4A**). Given the established roles of ACC synthase (ACS) and ACC oxidase (ACO) in ethylene biosynthesis, we first comprehensively identified members of the ACO and ACS families in *M. laxiflora* and constructed phylogenetic trees to compare them with their *Arabidopsis thaliana* homologs (**Supplementary Figure 4**). Following this identification, transcriptomic analysis of all *ACS* and *ACO* homologs was performed. *MylACO1* exhibited marked transcriptional induction under waterlogging stress, while other genes exhibited insignificant change (**Figure 4B**). Subsequent qPCR analysis confirmed a steady up-regulation of *MylACO1* during a 36-h waterlogging treatment, with a 12-fold increase relative to the start of treatment (**Figure 4C**). Consistent with the transcriptional induction of *MylACO1*, ACO enzyme activity increased significantly in *M. laxiflora* under waterlogging stress (**Figure 4D**). Collectively, these data demonstrate that waterlogging stress triggers ethylene accumulation in *M. laxiflora*, primarily through *MylACO1*-mediated biosynthetic regulation.

### 3.5 MylERF3 binds to the promoter of *MylACO1* and activates its expression

**Figure 5:**
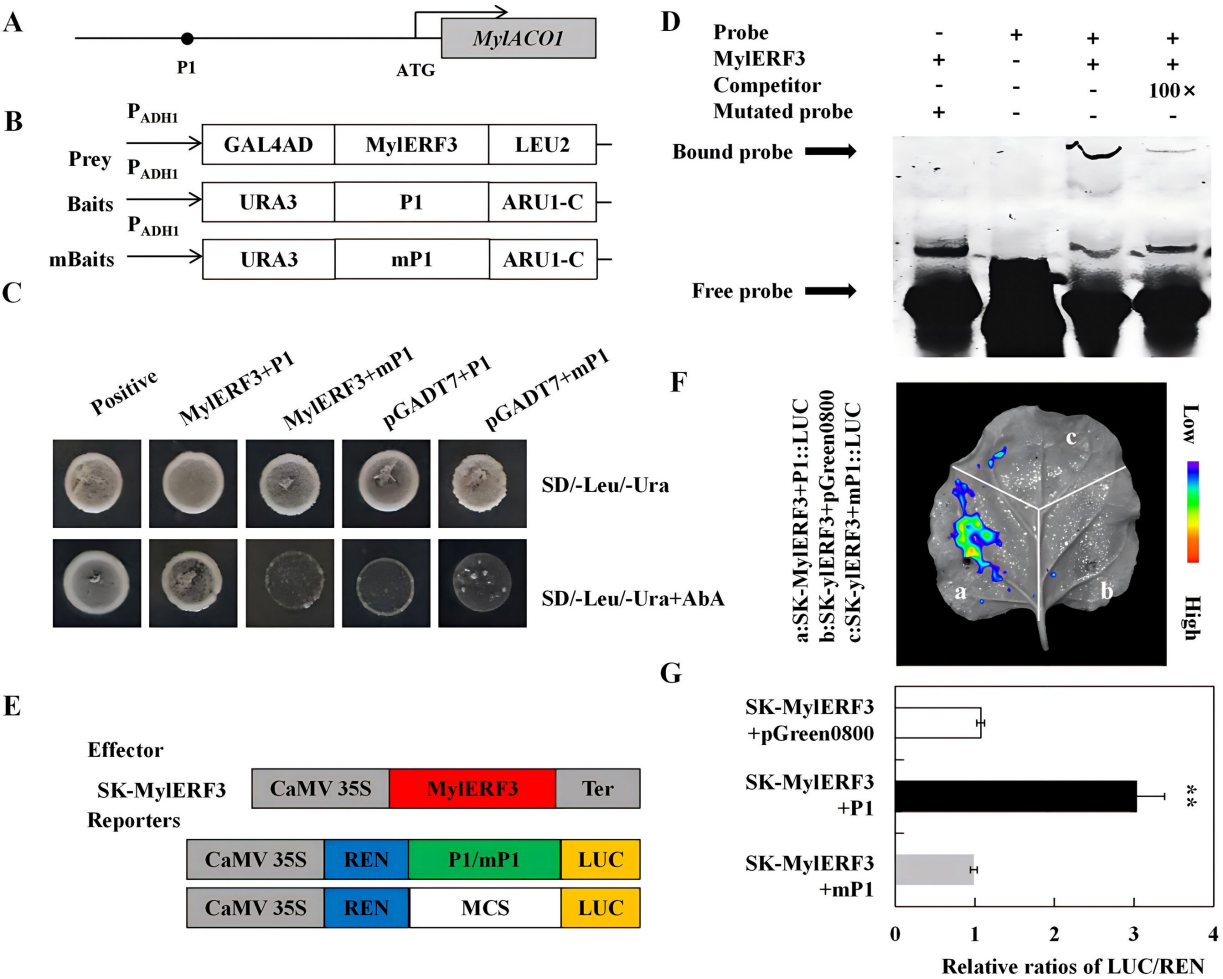
MylERF3 binds to and activates the promoter of *MylACO1*. **(A)** Schematic diagrams showing the *MylACO1* promoters. Black circles denote the positions of DRE/CRT motif. **(B)** Schematic representation of the prey and bait vectors used in Y1H assays. **(C)** Y1H assays showing the interaction between MylERF3 and the promoter of *MylACO1*. pGAD-p53 was used as a positive control. **(D)** EMSA demonstrating that MylERF3 specifically binds to *MylACO1* promoter. **(E)** Schematic illustration of constructs employed in dual-luciferase reporter assays. **(F, G)** Living image **(F)** and quantitative analysis **(G)** of dual-LUC transient expression assays of the promoter activity in tobacco. Error bars represent ± standard error (n=3). Asterisks indicate significant differences using Student’s *t*-test (**P < 0.01).

It has been suggested that ERFs play vital roles in ethylene signaling pathway to regulate abiotic stress responses. This compels us to investigate whether MylERF3 directly activates ethylene biosynthesis under waterlogging stress. ERF proteins can activate or repress transcription of genes by binding to GCC-box or DRE/CRT motifs in promoter regions (Nakano et al., 2006; Müller and Munné-Bosch, 2015; Yu et al., 2019). To identify potential binding sites of MylERF3, we analyzed the promoter sequence of *MylACO1* using the JASPAR database (https://jaspar.elixir.no/). This analysis identified a DRE/CRT motif GTCGG with the highest prediction score, suggesting it as a putative binding site for MylERF3 (**Figure 5A**).

To determine whether MylERF3 directly regulates *MylACO1* expression by binding to the DRE/CRT motif, a yeast one-hybrid (Y1H) assay was first performed with MylERF3 cloned into the prey vector (**Figure 5B**). All transformed yeast strains grew normally on SD/-Leu/-Ura medium, while only the positive control and yeast co-transformed with MylERF3 and the wild-type DRE/CRT motif (P1) grew on medium supplemented with AbA (**Figure 5C**). An electrophoretic mobility shift assay (EMSA) was subsequently conducted to verify the specific and direct binding between MylERF3 and the DRE/CRT motif within the P1 promoter fragment of *MylACO1*. The purified recombinant MylERF3 protein bound to the FAM-labeled probe, resulting in a visible gel shift. This binding was competitively inhibited in a dose-dependent manner by the addition of unlabeled probe. Furthermore, no shifted band was observed when MylERF3 was incubated with a labeled probe carrying a mutated DRE/CRT motif (**Figure 5D**). To further examine the *in vivo* interaction between MylERF3 and the *MylACO1* promoter, a dual-luciferase (LUC) reporter assay was conducted in *N. benthamiana* leaves (**Figure 5E**). The LUC/REN ratio was significantly higher in leaves co-expressing MylERF3-SK and the wild-type reporter than in those with empty effector controls (**Figure 5G**). Taken together, these results demonstrate that MylERF3 directly binds to DRE/CRT motif within the *MylACO1* promoter and activates its transcription.

### 3.6 Ectopic overexpression of *MylERF3* promotes ethylene production

**Figure 6:**
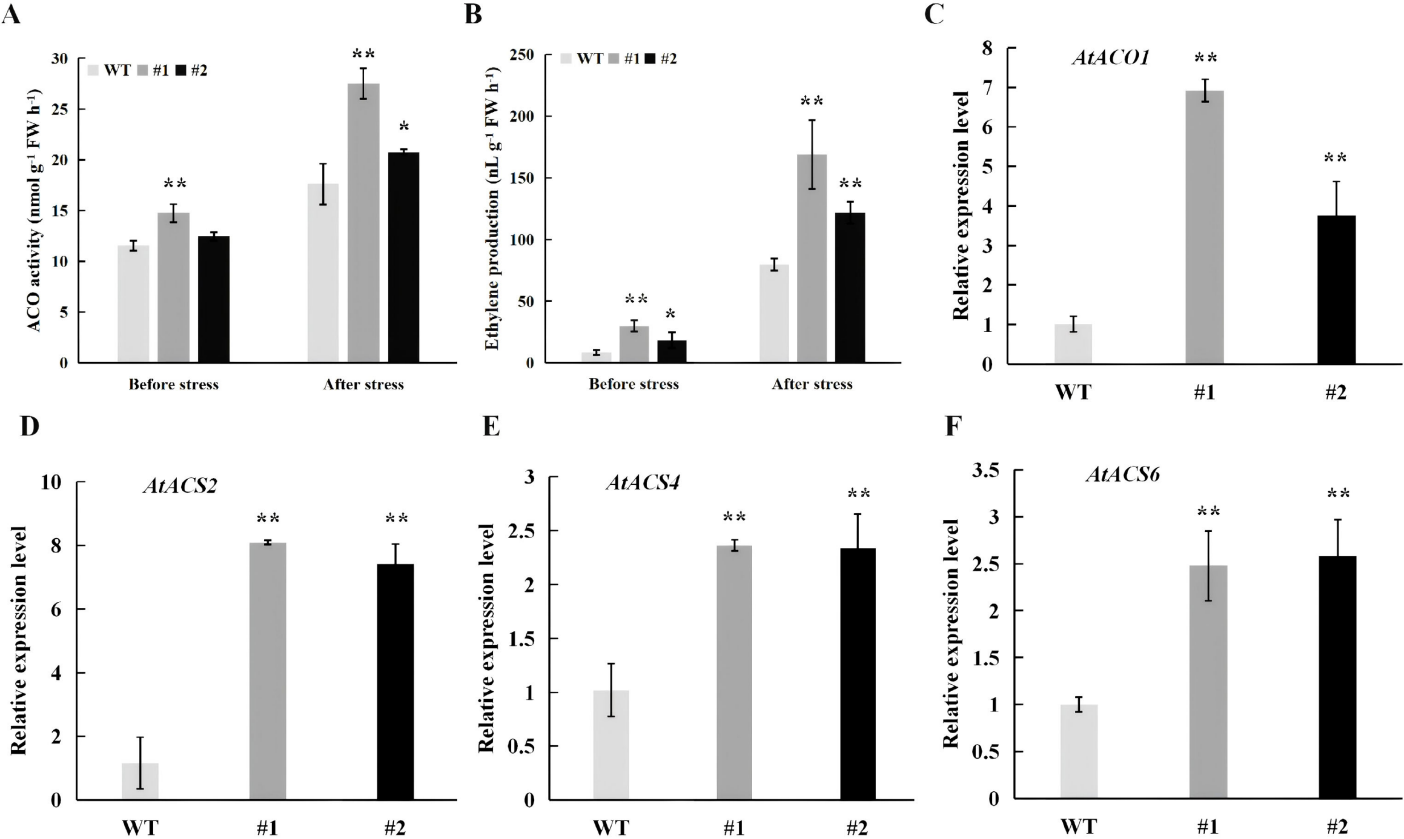
Overexpression of *MylERF3* promotes ethylene biosynthesis in *Arabidopsis*. **(A, B)** The ACO activity **(A)** and ethylene production **(B)** in two *MylERF3*-overexpression lines (#1 and #2) and the Col-0 wild type (WT). **(C–F)** The relative expression level of ethylene synthesis related genes in two *MylERF3*-overexpression lines (#1 and #2) and the Col-0 wild type (WT). Error bars represent ± standard error (n=3). Asterisks indicate significant differences using Student’s *t*-test (*P < 0.05, **P < 0.01).

Based on the observed interaction between MylERF3 and the *MylACO1* promoter, we hypothesized that MylERF3 enhances waterlogging tolerance by modulating ethylene biosynthesis. To verify this hypothesis, ethylene production and ACO enzyme activity were measured in wild-type (WT) and *MylERF3*-overexpressing transgenic *Arabidopsis* plants. The results revealed a significant increase in both ethylene levels and ACO activity in the transgenic lines compared to WT (**Figures 6A, B**). Consistently, the expression of ethylene biosynthesis-related genes (*AtACO1*, *AtACS2*, *AtACS4* and *AtACS6*) was also markedly up-regulated in the *MylERF3*-overexpressing lines (**Figures 6C–F**). These findings demonstrate that overexpression of *MylERF3* enhances waterlogging tolerance in *Arabidopsis* by promoting the ethylene biosynthesis pathway.

## 4 Discussion

Waterlogging stress severely constrains plant survival and productivity by causing hypoxia, which disrupts cellular energy homeostasis and results in the cytotoxic accumulation of metabolites and ROS (Xu et al., 2006; Sasidharan and Voesenek, 2015). Conversely, plants can acclimate to waterlogging stress through multiple adaptive mechanisms. Among these, transcriptional regulation mediated by waterlogging-responsive TFs and their target genes, plays a crucial role in enhancing waterlogging tolerance (Wei et al., 2019; Ateeq et al., 2023). ERF family TFs, in particular, have been identified as key regulators of the waterlogging response across various plant species. Nevertheless, the physiological and molecular mechanisms underlying the function of many ERFs remain poorly understood. In this study, we identified MylERF3, an AP2/ERF transcription factor in *M. laxiflora*, as a positive regulator of waterlogging tolerance by regulation of ethylene production via directly transcriptionally activating *MylACO1*. These results not only elucidate a key molecular module conferring the exceptional waterlogging adaptability of *M. laxiflora*, but also broaden our understanding of ethylene-mediated stress adaptation in plants.

The rapid upregulation of *MylERF3* under waterlogging conditions positions it as an early-response transcriptional regulator in flood adaptation. Current understanding of ERF-mediated waterlogging tolerance has been predominantly focused on the ERF-VII subfamily. For example, ERF-VII transcription factor AvERF75 positively regulates the waterlogging tolerance by interacting with *AvLOB41* in kiwifruit (Bai et al., 2023). *AtRAP2.2* in *Arabidopsis* (Hinz et al., 2010; Lou et al., 2022), *SUB1A* in rice (Xu et al., 2006; Fukao and Bailey-Serres, 2008; Fukao et al., 2011) and *ZmEREB180* in maize (Yu et al., 2019; Qi et al., 2025) are also upregulated under hypoxia and contribute to flood tolerance. In contrast, our phylogenetic result reveals that MylERF3 belongs to the ERF-III subfamily, indicating a divergent regulatory route underpinning waterlogging resilience and highlighting the functional diversity within the ERF family. Notably, the closest *Arabidopsis* homolog of MylERF3, AtERF41, has been implicated in salt and drought tolerance rather than in waterlogging adaptation (Wang et al., 2026). Similarly, the cotton ortholog GhERF41, which belongs to the ERF IIIe subfamily, has been reported to regulate fiber cell wall synthesis instead of stress responses, underscoring the functional divergence within this subfamily across species (Gao et al., 2023). Detailed sequence alignment further reveals that while the AP2/ERF DNA-binding domain of MylERF3 remains highly conserved, ensuring the recognition of target promoters. The pronounced variation in its N- and C-terminal domains likely underpins its specialized role in waterlogging adaptation. We propose that these divergent regulatory domains may have evolved to facilitate unique protein-protein interactions or upstream signaling recruitment, enabling MylERF3 to specifically orchestrate the submergence response in the riparian habitat of *M. laxiflora*. Within the ERF-III clade, the closest functional analog of MylERF3 is CmTINY2, a nuclear activator that enhances waterlogging tolerance by controlling stomatal aperture in chrysanthemum (Gu et al., 2024). These findings collectively demonstrate that MylERF3 represents a previously unrecognized regulatory pathway for flood adaptation, expanding the functional diversity of the ERF family beyond the well-characterized ERF-VII subfamily and highlighting the evolutionary diversification of ERF-III members across species.

Ethylene is a well-known mediator of plant responses to waterlogging, promoting aerenchyma formation, adventitious root development, and ROS scavenging (Sasidharan and Voesenek, 2015; Zhao et al., 2021; Atiq et al., 2026). Ethylene biosynthesis under stress conditions is primarily governed by the sequential actions of ACS and ACO. In the ethylene biosynthesis pathway, ACO is responsible for the final conversion of ACC to ethylene. In the present study, waterlogging treatment significantly upregulated *MylACO1* expression and enhanced total ACO enzyme activity in *M. laxiflora*. These changes coincided with a rapid ethylene burst, suggesting that *MylACO1* is a major contributor to waterlogging-induced ethylene production. This regulatory mechanism is consistent with findings in other species. For example, recent studies have demonstrated that waterlogging rapidly upregulates *ACO* and *ACS* homologs to trigger ethylene accumulation in *Brassica* species (Combs-Giroir and Gschwend, 2024). In flooded maize, adaptive aerenchyma formation and root remodeling critically depend on hypoxia-triggered ethylene synthesis (Geng et al., 2023). Therefore, the rapid activation of *MylACO1* serves as a core mechanism for *M. laxiflora* to generate the ethylene signal required for flood survival.

Although ethylene acts as a pivotal signaling molecule during waterlogging stress, its hyperaccumulation leads to premature senescence and cell death. Consequently, maintaining ethylene homeostasis is crucial for waterlogging survival (Müller and Munné-Bosch, 2015). In typical flood-tolerant models, this balance is strictly governed by specific transcriptional modules. For instance, waterlogging-induced ethylene in rice triggers the *SUB1A* pathway to restrict gibberellin-mediated elongation and enhance anaerobic metabolism, effectively conserving energy reserves (Xu et al., 2006; Fukao and Bailey-Serres, 2008). Upon waterlogging, ERF-VII proteins escape N-end rule degradation, thereby coordinating anaerobic gene expression and fine-tuning ethylene sensitivity to prevent tissue damage (Gibbs et al., 2011; Licausi et al., 2011; Gasch et al., 2016). In our study, a similar ethylene-dependent mechanism was observed in *M. laxiflora*. Waterlogging treatment significantly increased ethylene production by upregulating *MylACO1*. Specifically, the MylERF3 transcription factor targets the *MylACO1* promoter to activate its transcription. Furthermore, the overexpression of *MylERF3* in *Arabidopsis* resulted in enhanced ACO activity and elevated ethylene levels under waterlogging stress. These results elucidate that MylERF3 confers enhanced flood tolerance by acting as a transcriptional activator of ethylene biosynthesis.

Ecologically, the MylERF3-regulated ethylene accumulation is essential for the adaptation of *M. laxiflora* to its riparian habitat. During waterlogging, this module induces a “quiescence” response. However, the plant does not simply shut down its metabolism. Instead, it actively represses shoot elongation to conserve carbohydrates. The saved energy is then diverted to form aerenchyma and adventitious roots. Functionally, this physiological adaptation is highly analogous to the *SUB1A*-mediated survival strategy in flooded rice (Fukao and Bailey-Serres, 2008). In contrast, it differs from the “escape” strategy of deepwater rice, where *SNORKEL* genes stimulate rapid internode growth to reach the water surface (Hattori et al., 2009). Therefore, these results demonstrate that MylERF3 is required to establish the quiescence survival strategy under waterlogging stress in *M. laxiflora*.

Plant adaptation to waterlogging relies heavily on the coordination between ethylene signaling and ROS scavenging (Steffens, 2014; Sasidharan et al., 2018; Asim et al., 2025). Our results show that transgenic *Arabidopsis* expressing *MylERF3* maintained lower ROS levels (H_2_O_2_ and O_2_^•-^) during waterlogging, suggesting a role for MylERF3 in mitigating oxidative damage. Parallel to our findings, previous transcriptomic profiling of flooded *M. laxiflora* revealed the strong induction of major antioxidant enzymes (Li et al., 2022; Wang et al., 2024). A comparable dual-regulatory mechanism was recently reported in *Poncirus trifoliata*, where the ERF transcription factor PtrERF9 enhances cold tolerance by directly activating both an ethylene biosynthesis gene *PtrACS1* and an antioxidant gene *PtrGSTU17* (Zhang et al., 2022b). These observations imply that MylERF3 may also regulate the antioxidant defense network. This regulation could be an indirect consequence of ethylene accumulation, or a direct result of MylERF3 binding to DRE/CRT elements in the promoters of antioxidant genes. Validating the direct transcriptional targets of MylERF3 within the ROS-scavenging pathway will be the focus of our future studies.

Additionally, the functional characterization through heterologous overexpression in *Arabidopsis* is a limitation of this study, as a stable transformation system for *M. laxiflora* is not yet available. *Arabidopsis* was an appropriate heterologous system because the ethylene biosynthesis pathway is largely conserved between different plant species, and its utility for functional dissection of ERF transcription factors from non-model species has been repeatedly demonstrated. Whole-plant experiments in the native species, such as CRISPR/Cas9-mediated knockout or complementation, will be necessary to definitively validate the MylERF3-MylACO1 module. Continued efforts to develop genetic transformation in this woody riparian species are therefore required to complete the functional analysis.

## 5 Conclusions

Based on these findings, we propose a working model for MylERF3-mediated waterlogging tolerance in *M. laxiflora*. Waterlogging stress rapidly induces the expression of *MylERF3*, which subsequently activates *MylACO1* transcription through direct binding to its promoter region. Consequently, the enhanced ACO enzyme activity drives a massive accumulation of ethylene. This ethylene signaling module, likely coordinating with other downstream survival networks, ultimately confers robust waterlogging tolerance. Furthermore, this model broadens our understanding of how riparian plants transcriptionally reprogram ethylene biosynthesis to survive waterlogging. Ultimately, we identify MylERF3 as a critical positive regulator of flood adaptation, functioning primarily through the targeted activation of *MylACO1* to stimulate ethylene production.

## Data Availability

The roots of *M. laxiflora* under waterlogging stress RNA-seq data were downloaded from NCBI database (PRJNA840865). The data that support the findings of this study are available from the corresponding author upon reasonable request.
