# Supplementary material for: ERF3 of Myricaria laxiflora contributes to waterlogging tolerance by regulating ethylene biosynthesis through transcriptional regulation of MylACO1
Source: Front Plant Sci. 2026 Jul 30;17:1851263. doi: 10.3389/fpls.2026.1851263 (PMC13470107; doi:10.3389/fpls.2026.1851263)
Supplement: Supplementary file 1 [file DataSheet1.docx]

**Figure S1. Phylogenetic analysis and multiple alignments of MylERF3 with *Arabidopsis thaliana*. (A)** A phylogenetic tree was constructed based on the amino acid sequences of MylERF3 with all ERF gene from *Arabidopsis thaliana* by using MEGA 7.0 software based on the neighbour-joining method and bootstrap analysis with 1000 replications. (**B)** Multiple alignments were performed with GENEDOC software using the conserved domain of MylERF3 with *Arabidopsis* orthologs.

**Figure S2. Molecular identification of *MylERF3*-overexpressing Arabidopsis thaliana lines. (A)** The transformed vectors were exampled by PCR using genomic DNA samples. M: DNA marker; P, plasimid. (B) qRT-PCR was used to analyze the *MylERF3* expression of transgenic *Arabidopsis*. Error bars indicate ± SE (n = 3). Asterisks indicate significant difference between WT and transgenic *Arabidopsis* (one-way ANOVA with Tukey's test, ***P* < 0.01).

**Figure S3. Relative expression levels of antioxidant genes in *MylERF3*-overexpressing Arabidopsis lines. (A-C)** Expression patterns of *AtSOD1* **(A)**, *AtCAT1* **(B)** and *AtAPX1* **(C)** in the WT and two independent transgenic lines (#1 and #2). Error bars represent ± standard error (n=3). Asterisks indicate significant differences using Student’s *t*-test (** *P*<0.01).

**Figure S4. Phylogenetic analysis of the ACO and ACS protein families. (A)** Phylogenetic tree of ACO proteins from *M. laxiflora* and *Arabidopsis thaliana*. **(B)** Phylogenetic tree of ACS proteins from *M. laxiflora* and *Arabidopsis thaliana*.
